# The Brain in Oral Clefting: A Systematic Review With Meta-Analyses

**DOI:** 10.3389/fnana.2022.863900

**Published:** 2022-06-10

**Authors:** Kinga A. Sándor-Bajusz, Asaad Sadi, Eszter Varga, Györgyi Csábi, Georgios N. Antonoglou, Szimonetta Lohner

**Affiliations:** ^1^Department of Pediatrics, University of Pécs, Pécs, Hungary; ^2^Doctoral School of Clinical Neurosciences, University of Pécs, Pécs, Hungary; ^3^Adult Psychiatric Division, Borlänge Specialist Clinic, Borlänge, Sweden; ^4^Periodontology Unit, Faculty of Dentistry, Centre for Host Microbiome Interactions, Oral and Craniofacial Sciences, King’s College London, London, United Kingdom; ^5^Cochrane Hungary, Clinical Centre of the University of Pécs, Medical School, University of Pécs, Pécs, Hungary; ^6^Department of Public Health Medicine, Medical School, University of Pécs, Pécs, Hungary

**Keywords:** cleft lip, cleft palate, neurodevelopment, brain, neuroimaging

## Abstract

**Background:**

Neuroimaging of individuals with non-syndromic oral clefts have revealed subtle brain structural differences compared to matched controls. Previous studies strongly suggest a unified primary dysfunction of normal brain and face development which could explain these neuroanatomical differences and the neuropsychiatric issues frequently observed in these individuals. Currently there are no studies that have assessed the overall empirical evidence of the association between oral clefts and brain structure. Our aim was to summarize the available evidence on potential brain structural differences in individuals with non-syndromic oral clefts and their matched controls.

**Methods:**

MEDLINE, Scopus, Cochrane Central Register of Controlled Trials, Web of Science and Embase were systematically searched in September 2020 for case-control studies that reported structural brain MRI in individuals with non-syndromic oral clefts and healthy controls. Studies of syndromic oral clefts were excluded. Two review authors independently screened studies for eligibility, extracted data and assessed risk of bias with the Newcastle-Ottawa Scale. Random effects meta-analyses of mean differences (MDs) and their 95% confidence intervals (95% CI) were performed in order to compare global and regional brain MRI volumes.

**Results:**

Ten studies from 18 records were included in the review. A total of 741 participants were analyzed. A moderate to high risk of bias was determined for the included studies. The cerebellum (MD: −12.46 cm^3^, 95% CI: −18.26, −6.67, *n* = 3 studies, 354 participants), occipital lobes (MD: −7.39, 95% CI: −12.80, −1.99, *n* = 2 studies, 120 participants), temporal lobes (MD: −10.53 cm^3^, 95% CI: −18.23, −2.82, *n* = 2 studies, 120 participants) and total gray matter (MD: −41.14 cm^3^; 95% CI: −57.36 to −24.92, *n* = 2 studies, 172 participants) were significantly smaller in the cleft group compared to controls.

**Discussion:**

There may be structural brain differences between individuals with non-syndromic oral clefts and controls based on the available evidence. Improvement in study design, size, methodology and participant selection could allow a more thorough analysis and decrease study heterogeneity.

## Introduction

Oral clefts are one of the most common birth defects with a worldwide incidence of 1:700 births (Mossey and Modell, 2012). Oral clefts can be syndromic or non-syndromic, the latter occurring as a single anomaly in the absence of other physical and developmental disorders (Mossey and Modell, 2012; Bjørnland et al., 2021). The etiology of oral clefts is multifactorial, including gene-environmental interactions, hereditary causes, antenatal nutrition, and drug exposure (Lithovius et al., 2014; Bjørnland et al., 2021). Oral clefts can be anatomically classified as cleft lip (CL), cleft palate (CP), and combined cleft lip and palate (CLP) (Lithovius et al., 2014; Bjørnland et al., 2021).

Syndromic oral clefts are predisposed to more complex treatment due to the underlying genetic disorder and other associated health complications (Sándor-Bajusz et al., 2021). Syndromic individuals often have mental comorbidities including intellectual disability and learning disorders (Hardin-Jones and Chapman, 2011; Diaz-Stransky and Tierney, 2012; Feragen et al., 2014; McDonald-McGinn et al., 2015; Zinkstok et al., 2019). Decades of research revealed the presence of neuropsychiatric and neurodevelopmental disorders in individuals with non-syndromic oral clefts (Broder et al., 1998; Richman and Ryan, 2003; Conrad et al., 2008; Pedersen et al., 2016; Ansen-Wilson et al., 2018; Tillman et al., 2018). Children with oral clefts are associated with a significant agglomeration of psychiatry disorders including intellectual disability, autism spectrum disorder, ADHD and learning disorders (Pedersen et al., 2016; Ansen-Wilson et al., 2018; Tillman et al., 2018). Neurodevelopmental delays have been documented in younger children including fine motor, gross motor and both expressive and receptive language development (Conrad et al., 2008; Hardin-Jones and Chapman, 2011; Gallagher and Collett, 2019). These observations were suggested to be the consequence of multiple stressors including social stigma, frequent anesthesia exposure and/or cleft-related airway obstruction impairing proper neurodevelopment (Gallagher and Collett, 2019).

New advances in oral cleft research have strongly suggested a unified primary dysfunction of normal brain and face development, that could explain the neurodevelopmental-related deficits observed in these children (Conrad et al., 2021). This primary dysfunction seems to affect a crucial developmental stage of a physiological migration of cells that will later form the face and parts of the brain and the central nervous system (Ansen-Wilson et al., 2018; Ornoy, 2020). Neuroimaging studies have additionally revealed significant differences in the brain structure of individuals with non-syndromic oral clefts compared to matched controls. However, a definitive statement cannot be made due to the heterogeneity among the studies including quality, sample size, methodology and outcomes (Yang et al., 2012; Gallagher and Collett, 2019).

The aim of the present systematic review was to assess the overall empirical evidence of the association between of non-syndromic oral clefts and the brain.

## Methods

The current meta-analysis was registered in PROSPERO (International Prospective Register of Systematic Reviews^1^; RRID:SCR_019061, identifier CRD42020167773), and is reported according to the Preferred Reporting Items for Systematic Reviews and Meta-Analyses 2020 (PRISMA 2020, RRID:SCR_018721) guideline (Page et al., 2021).

### Search Strategy

Searches of the following databases were conducted until 7 September 2020: MEDLINE (Ovid; RRID:SCR_002185), Scopus, Cochrane Central Register of Controlled Trials (CENTRAL; RRID:SCR_006576), Web of Science and Embase (RRID:SCR_001650). Clinicaltrials.gov (RRID:SCR_002309) was searched to identify ongoing/completed studies and unpublished SRs (see Supplementary Table 1 for the full search strategy used in each of the databases).

### Selection of Studies

#### Inclusion Criteria

The following criteria had to be met for inclusion into the study: (1) Case-control studies with humans; (2) Individuals with non-syndromic (isolated) oral clefts, without restriction to age; (3) Healthy controls; (4) Structural brain differences of individuals with non-syndromic oral clefts vs. their controls as a relevant outcome: structural differences had to be explored with brain MRI. No restrictions were applied for language.

#### Exclusion Criteria

The publication was excluded if it had any of the following: (1) Animal studies (2) Individuals with syndromes (syndromic forms of oral clefts, such as Pierre-Robin sequence or Velocardiofacial syndrome).

The selection process was performed with the Covidence systematic review software (RRID:SCR_016484) (Veritas Health Innovation, 2017).

Two review authors (KSB and EV) screened the titles and/or abstracts of studies retrieved from the searches. Additional sources were also screened (hand searching, reference/citation lists) to identify articles that may potentially meet the inclusion criteria. Full texts of these potentially eligible records were retrieved and assessed by one review author (KSB), while a second checked the decisions (EV). Any differences between the two reviewers were settled by consensus after consulting a third author (GA or SL).

### Data Extraction

Data was extracted independently by three authors (KSB, AS, and EV). Discrepancies were resolved the same way as stated above.

Study setting (design, institution, country), patient demographics (number, age, sex, ethnicity, gender, type of oral cleft, brain imaging details, data processing) and outcome measurement details (general and regional brain MRI measurements) were collected. Any data that were not described in the article were calculated from existing data, or were obtained by contacting the authors.

The primary outcome measures were structural differences of the brain of individuals with oral clefts vs. individuals without oral clefts (controls) investigated *via* MRI. Other sought outcomes included the correlation between observed structural differences in the brain of individuals with oral clefts and alterations in neurological and/or mental functioning compared to controls.

### Risk of Bias Assessment

The Newcastle-Ottawa Scale (NOS) (Wells et al., 2000) was used for all outcomes to assess the quality of non-randomized case-control studies included in the systematic review. Assessment was completed by two authors (KSB, AS) and independently checked by a third (SL) the same way to resolve discrepancies.

### Statistical Analysis and Data Synthesis Methods

Review Manager Software Version 5.4 was used for data synthesis (RRID:SCR_003581) (Cochrane, 2020). The random-effects model was chosen *a priori* as the primary method to estimate all pooled estimates for studies that were comparable in design, exposure and outcomes. This model was used to account for the differences within study populations such as age, sex, and type of oral clefts. Mean Differences (MDs) and their corresponding 95% confidence intervals (CI 95%) were used for continuous outcomes.

The extent and impact of between-study heterogeneity was assessed by inspecting the forest plots and by calculating the tau-squared and the I-squared statistics, respectively. The I-squared thresholds represented heterogeneity that may not be important (0–40%), moderate (30–60%), substantial (50–90%), or considerable (75–100%). Possible sources of heterogeneity in meta-analyses were sought through pre-specified mixed-effects subgroup analyses if at least two studies were included for a comparison (same intervention/outcome). Pre-defined subgroup analyses included: (i) age; (ii) sex; (iii) ethnicity; (iv) cleft form (non-syndromic vs. syndromic).

#### Additional Analyses

Assessment of reporting biases (small-study effects or publication bias) was planned through the inspection of a contour-enhanced funnel plot and with the Egger’s weighted regression test if a sufficient number of trials were identified (*n* > 10).

## Results

### Study Selection (Systematic Literature Search)

A total of 257 records were identified following the database searches. Overall, 245 records underwent title and abstract screening following duplicate removal. Thirty-two records were retrieved and assessed for eligibility. Two records were additionally identified by handsearching, and only one met the inclusion criteria (Yang et al., 2012). A total of 10 studies from 18 records met the inclusion criteria. Three records included individuals diagnosed with Van der Woude syndrome, a syndromic form of oral clefts (Nopoulos et al., 2000, 2002, 2005). These records were included in the current systematic review as none of the syndromic individuals exceeded 15% of total cleft participants.

**FIGURE 1:**
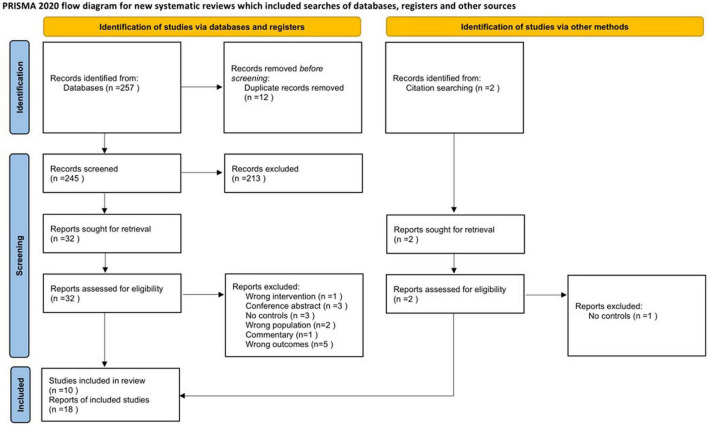
Flow diagram of the study selection process.

The study selection process is shown in the flow diagram of Figure 1.

Fifteen records seemed to meet the inclusion criteria, however, they were excluded during the full-text screening process. The reasons for exclusion were as follows: absence of a control group (*n* = 3 Shen and Huang, 1996; Mueller et al., 2007; Zheng et al., 2019), conference abstracts or commentaries (*n* = 4 Chollet et al., 2010; Tollefson and Sykes, 2010; DeVolder et al., 2014, 2015), wrong study population that only included syndromic cases of oral clefts (*n* = 2 Nopoulos et al., 2007a,b), absence of neuroimaging (*n* = 5 Čeponiene et al., 1999; Scott et al., 2005; Kummer et al., 2007; Conrad et al., 2008; Watkins et al., 2018), or neuroimaging other than brain MRI (*n* = 1 Becker et al., 2008).

#### Study Characteristics

The study characteristics are presented in Tables 1A,B. The majority were conducted in the US. Other countries included Australia (Adamson et al., 2014), Brazil (Bodoni et al., 2021), and China (Yang et al., 2012; Li et al., 2020). Study size ranged between 24 and 234 participants. The majority of the participants were males of Caucasian ethnicity. Most of the participants were children.

**TABLE 1A:** Characteristics of included studies.

References	Country	Study participants present in another reference?	Inclusion	Exclusion	N
Nopoulos et al. (2000)	United States	No	Adult males (18 +) with non-syndromic oral clefts	Congenital syndromes	28
Nopoulos et al. (2001)	United States	No	Adult males with non-syndromic oral clefts	Congenital syndromes	124
Nopoulos et al. (2002)	United States	No	Non-syndromic oral clefts	Congenital syndromes	92
Nopoulos et al. (2005) (Nopoulos, 2002A)	United States	Same study cohort as (Nopoulos et al., 2002)	Adult males (18 +) with non-syndromic clefts	Congenital syndromes	92
Shriver et al. (2006) (Nopoulos, 2002B)	United States	Same patient population as (Nopoulos et al., 2002)	Adult males (18 +) with non-syndromic oral clefts	Genetic syndrome, serious, active medical or neurologic disease or active substance abuse/dependence, psychiatric disorders	89
Nopoulos et al. (2007c)	United States	No	Children with non-syndromic oral clefts	Braces (artifact in MRI scan), IQ < 70, genetic syndrome	148
Boes et al. (2007) (Nopoulos, 2007A)	United States	Subset of cleft participants from Nopoulos et al. (2007c)	Boys with non-syndromic oral clefts	Genetic syndromes, serious medical or neurological disease	73
Weinberg et al. (2009)	United States	No	Adult males (18 +)	N/A	86
van der Plas et al. (2010) (Nopoulos, 2007E)	United States	Participants of both groups were part of another study (Nopoulos et al., 2007c)	Children with unilateral CLP or CL only	CP, bilateral CLP or CL, genetic syndromes, serious medical and neurological disease	90
Nopoulos et al. (2010) (Nopoulos, 2007B)	United States	Subset of cleft participants from Nopoulos et al. (2007c)	Boys with non-syndromic oral clefts	Braces (creates artifact in MRI scan), IQ < 70, genetic syndrome	110
Conrad et al. (2010) (Nopoulos, 2007C)	United States	Cleft MRI results from Nopoulos et al. (2007c)	Children with non-syndromic oral clefts	Genetic syndromes, significant hearing loss (requiring a hearing aid), braces, history of head trauma, brain tumor or epilepsy.	86
DeVolder et al. (2013) (Nopoulos, 2007D)	United States	Subset of participants of two previous studies from Nopoulos et al. (2007c) and Conrad et al. (2010)	Children with non-syndromic oral clefts	Braces (artifact in MRI scan), IQ < 70	234
Yang et al. (2012)	China	No	Full-term birth, uncomplicated delivery, non-syndromic oral cleft	Congenital syndromes, other chronic health disorders	54
Weinberg et al. (2013)	United States	No	Males, non-syndromic oral clefts, limited to 18–50 year old	Congenital syndromes	64
Adamson et al. (2014)	Australia	No	Children with non-syndromic oral clefts	Genetic syndromes	52
Chollet et al. (2014) (Nopoulos, 2007F)	United States	MRI data from previous study by Nopoulos et al. (2007c)	Children with non-syndromic oral clefts	Braces, FSIQ < 70, genetic syndromes	96
Bodoni et al. (2021)	Brazil	No	Children with non-syndromic oral clefts	Sensory or motor problems, psychiatric disorders, claustrophobia, contraindications to MRI	24
Li et al. (2020)	China	No	N/A	Brain structural abnormalities, neurological or psychiatric disorders, and MRI contraindications	69

*N, population size; CLP, Cleft lip and palate; CP, Cleft palate; CL, Cleft lip.*

**TABLE 1B:** Demographic data of included studies.

References		Demographic measures of clefts			Demographic measures of controls		
	Age: mean (SD)	Gender (%)	Ethnicity (%)	Cleft subtype (N)	Age: mean (SD)	Gender (%)	Ethnicity (%)
Nopoulos et al. (2000)	33.7 (7.3)	Male (100%)	Caucasian (100%)	CL (1), CPO (5, one is syndromic), CLP (8, one is syndromic)	33.1 (7.7)	Male (100%)	Caucasian (100%)
Nopoulos et al. (2001)	30.3 (N/A)	Male (100%)	Caucasian (100%)	CPO (15), CLP (34, three are syndromic)	27.3 (N/A)	Male (52%), female (48%)	N/A
Nopoulos et al. (2002)	30.1 (7.04)	Male (100%)	Caucasian (100%)	CPO (14), CLP (32, three are syndromic)	28.8 (7.60)	Male (100%)	Caucasian (100%)
Nopoulos et al. (2005) (Nopoulos, 2002A)	30.1 (7.04)	Male (100%)	Caucasian (100%)	CPO (14), CLP (32, three are syndromic	28.8 (7.60)	Male (100%)	Caucasian (100%)
Shriver et al. (2006) (Nopoulos, 2002B)	30.1 (7.04)	Male (100%)	Caucasian (100%)	CPO (14), CLP (32, three are syndromic)	28,8 (7.60)	Male (100%)	Caucasian (100%)
Nopoulos et al. (2007c)	12.1 (3.26)	Male (67.57%), female (33.33%)	White (90.5%), Asian American (8, 1%), Hispanic (1.4%)	CL (18), CPO (23), CLP (33)	12.3 (3.08)	Male (67.57%), female (33, 33%)	White (87.8%), Asian American (5.4%), Hispanic (6.8)
Boes et al. (2007) (Nopoulos, 2007A)	9.98 (1.64)	Male (100%)	Provided for both study groups: African (1.37%), Asian (1.37%), Asian American (4.11%), Caucasian (89.04%), Hispanic (1,37%), and mixed (2.74%).	CL (8), CPO (7), CLP (15)	10.68 (1.45)	All male	See oral cleft group
Weinberg et al. (2009)	30.1 (7.1)	Male (100%)	Caucasian (100%)	CPO (14), CLP (31)	28.8 (7.5)	All male	Caucasian (100%)
van der Plas et al. (2010) (Nopoulos, 2007E)	Separated by cleft side: Right, 13 (2.68); left cleft, 11.7 (2.80)	Male (100%)	N/A	CL (9), CLP (24)	12,2 (3.01)	All males	N/A
Nopoulos et al. (2010) (Nopoulos, 2007B)	11.9 (3.3)	Male (100%)	Caucasian (95%; detailed info N/A)	CL (11), CPO (13), CLP (26)	12.1 (2.7)	All males	See oral cleft group
Conrad et al. (2010) (Nopoulos, 2007C)	13.27 (3.28)	Male, (59%) female (41%)	White (70%) Asian American (9%), Hispanic (5%), multiracial (7%) unknown (9%)	CL (7), CPO (11), CLP (25)	13.28 (3.27)	Males (59%), females, (41%)	White: 37 (86%), multiracial: 1 (2%), unknown: 5 (12%)
DeVolder et al. (2013) (Nopoulos, 2007D)	Male: 13.44 (4.61), female: 14.11 (3.80)	Male: (61.68%). female: (38.31%)	N/A	CL (22), CP (31), CLP (52)	Male: 13.04 (3.92), female: 13.65 (3.82)	Males (50.39%), females: 63 (49.60%)	N/A
Yang et al. (2012)	15.6 months (5.7 months)	Male: 24 (88.9%), female: 3 (11.1%)	Han Chinese (100%)	CL (2), CP (6), CLP (19)	15.6 months (5.7 months)	Same as oral cleft group	Han Chinese (100%)
Weinberg et al. (2013)	32.3 (7.4)	All male	N/A	N/A	29.1 (7.9)	All male	N/A
Adamson et al. (2014)	10.40 (2.57)	Males: 11 (42.31%) Females: 15 (57.69%)	N/A	N/A	10, 52 (1.72)	Male (61, 54%), female (38.46%)	N/A
Chollet et al. (2014) (Nopoulos, 2007F)	CP: 11.7 (± 3.2), CLP: 12.7 (± 3.1)	Male (66, 67%), female (33, 33%)	Caucasian (82%), Asian American (8%), African American (1%), Hispanic/Latino (2%), Native Hawaiian/Pacific Islander (1%), biracial (4%), N/A (1%)	CP (22), CLP (35)	12.5 (3.0)	Male (69.23%) female (30.77%)	See oral cleft group
Bodoni et al. (2021)	13 (1)	Male (58, 33%), female (41, 67%)	N/A	CLP (12)	13 (2)	Male (58.33%), female (41.67%)	N/A
Li et al. (2020)	Group B before therapy: 24 (4.92)*, group A after therapy 22.8 (5.4)*	Male: 26 (57.78%) female:19 (42.22%)	N/A	N/A	22 (1.58)*	Male: 15 (62.50%), female: 9 (37.50%)	N/A

N, population size; CLP, Cleft lip and palate; CP, Cleft palate; CL, Cleft lip. *Data were calculated from median (IQR) values with statistical tool developed by Wan et al. (2014) and Luo et al. (2018).

### Risk of Bias of Included Studies

The risk of bias assessment of included studies are shown in Table 2. The overall risk of bias ranged from medium to high. Selection of cleft participants, their comparators and the assessment of exposure were described in half of the studies. Information on recruitment and reasons for dropout were not available in most studies. Only one study reported blinding personnel of group status during MRI scanning (Nopoulos et al., 2007c).

**TABLE 2:** Risk of bias (RoB) assessment using the Newcastle-Ottawa Scale.

Studies	Selection				Comparability	Outcome			Total quality score
Author, year	Is the case definition adequate?	Representativeness of the cases	Selection of controls	Definition of controls	Comparability of cases and controls on the basis of design or analysis	Ascertainment of outcome	Same method of ascertainment for cases and controls	Non-response rate	9 = Low RoB; 7–8 = Medium RoB; < 6 = High RoB
Nopoulos et al. (2000)	*	*	*	*	**	*	*	*	**6**
Nopoulos et al. (2001)	*	*	*	*	**	*	*	*	**5**
Nopoulos et al. (2002)	*	*	*	*	**	*	*	*	**7**
Nopoulos et al. (2007c)	*	*	*	*	**	*	*	*	**8**
Weinberg et al. (2009)	*	*	*	*	**	*	*	*	**5**
Yang et al. (2012)	*	*	*	*	**	*	*	*	**7**
Weinberg et al. (2013)	*	*	*	*	**	*	*	*	**6**
Adamson et al. (2014)	*	*	*	*	**	*	*	*	**8**
Bodoni et al. (2021)	*	*	*	*	**	*	*	*	**7**
Li et al. (2020)	*	*	*	*	**	*	*	*	**4**

*Total quality score of 9 indicates low RoB, 7–8 medium RoB and ≤ 6 high RoB (Wells et al., 2000; Muka et al., 2020). The asterisks represent the scores under each dimension of the Newcastle-Ottawa Scale.*

### Results

Five studies were comparable in terms of study design, exposure and outcome. Studies were pooled using a random-effect meta-analysis.

All five studies segmented the brain according to all or one of the following: intracranial volume was divided into total brain tissue and cerebrospinal fluid; the brain tissue was divided into the cerebrum and cerebellum; the cerebrum was subdivided into the frontal, parietal, temporal, and occipital lobes. The majority of the studies used the Talairach Atlas-based method for measures of general and regional brain tissue. Most studies used three different sequences (T1-weighted, T2-weighted, and/or proton density images) with comparable parameters to classify tissue into gray matter, white matter, and cerebrospinal fluid. Additional details of MRI analysis are presented in Supplementary Table 2.

#### Primary Outcome

##### Studies Investigating Global Measurements

Global measurements were anatomically grouped into three groups: total brain volumes (including MRI volumes of the cerebrum and cerebellum), cerebral volumes (only MRI volumes of the cerebrum), and cerebellar volumes (only MRI volumes of the cerebellum).

###### Total Brain Volumes

**FIGURE 2:**
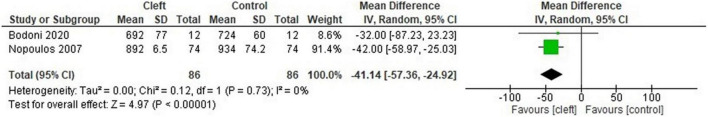
Forest plot for total brain gray matter volume (cm^3^).

**FIGURE 3:**
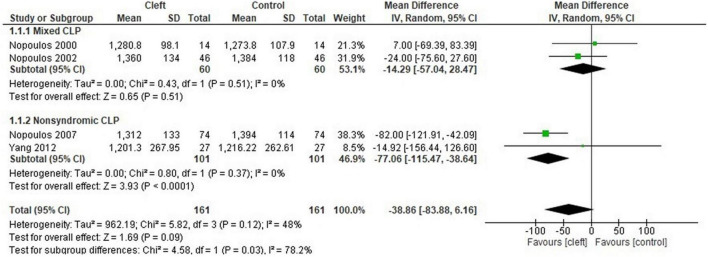
Forest plot for total brain volume (cm^3^) with subgroup analysis (non-syndromic vs. mixed).

The cleft group had lower total gray matter volume compared to controls (MD: −41.14 cm^3^; 95% CI: −57.36 to −24.92; *n* = 2; 172 participants; I^2^: 0%) (Figure 2). There were no differences in brain size of oral cleft subjects compared to controls (MD: −38.86 cm^3^; 95% CI: −83.88 to 6.16; *n* = 4;322 participants; I^2^: 48%) (Figure 3). No differences were found in white matter volume of oral cleft subjects and their controls (MD: −21.93 cm^3^; 95% CI: −64.20 to 20.33; *n* = 2; 172 participants; I^2^: 69%) (see Supplementary Figure 1).

###### Cerebral Volume

**FIGURE 4:**
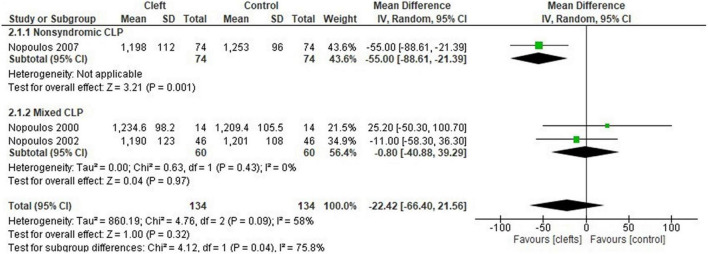
Forest plot for total volume of the cerebrum (cm^3^) with subgroup analysis (non-syndromic vs. mixed).

Total volume of the cerebrum in the oral cleft group did not differ from the control group (MD: −22.42 cm^3^; 95% CI: −66.40 to 21.56; *n* = 3; 268 participants; I^2^: 58%) (Figure 4). There were no differences in gray matter volume of the cerebrum between oral clefts and controls (MD: −6.45 cm^3^; 95% CI: −25.17 to 12.27; *n* = 2; 202 participants; I^2^: 0%) (see Supplementary Figure 2). An included study found a significantly lower gray matter volume on the left side of the cerebrum in individuals with oral cleft (Yang et al., 2012, *P* = 0.033). However, the study could not be included in the meta-analysis due to incomplete data (missing SD values). No differences were observed in cerebral white matter volume between oral clefts and controls (MD: −5.08 cm3; 95% CI: −20.19 to 10.03; *n* = 2; 146 participants; I^2^:0%) (Supplementary Figure 3).

###### Cerebellar Volume

**FIGURE 5:**
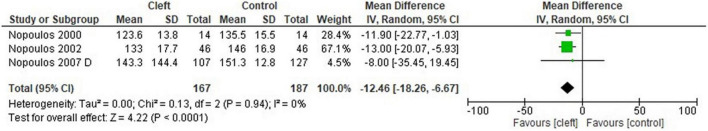
Forest plot for total volume of the cerebellum (cm^3^).

The cerebellum was significantly smaller in oral clefts compared to controls (MD: −12.46 cm^3^; 95% CI: −18.26, −6.67; *n* = 3; 354 participants; I^2^: 0%, *n* = 3) (Figure 5).

##### Studies Investigating Regional Measurements

###### Frontal Lobe Volume

The size of the frontal lobe did not differ between the cleft group and controls (MD: 18.27 cm^3^; 95% CI: −12.62 to 49.16; *n* = 2; 120 participants I^2^: 0%) (Supplementary Figure 4). There were no differences in frontal gray matter volume between oral clefts and controls (MD: 4.77 cm^3^; 95% CI: −7.84 to 17.38; *n* = 2; 165 participants; I^2^: 0%) (Supplementary Figure 5). There were no differences in the two components of the ventrofrontal cortex; the straight gyrus (MD: −0.17 cm^3^; 95% CI: −1.35 to 1.00; *n* = 2; 165 participants; I^2^: 90%) and orbitofrontal cortex (MD: −0.99 cm^3^; 95% CI: −2.69 to 0.71; *n* = 2; 165 participants; I^2^: 0%) (see Supplementary Figures 6, 7).

###### Parietal Lobe Volume

There were no differences in the size of the parietal lobe between the cleft group and controls (MD: 4.91 cm^3^; 95% CI: −4.29 to 14.10; *n* = 2; 120 participants; I^2^: 0%) (see Supplementary Figure 8).

###### Temporal Lobe Volume

**FIGURE 6:**
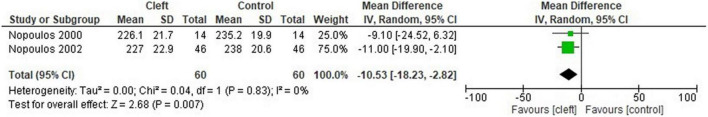
Forest plot for temporal lobe volume (cm^3^).

Smaller temporal lobes were found for the cleft group compared to controls (MD: −10.53 cm^3^; 95% CI: −18.23 to −2.82; *n* = 2; 120 participants; I^2^: 0%) (Figure 6). No differences were found on any side of the Superior temporal plane (STP) (left side MD: −0.37 cm3; −1.78 to 1.04; *n* = 2; 143 participants; I^2^: 66%. Right side MD: 0.20 cm3; 95% CI: −0.21 to 0.60; *n* = 2; 143 participants; I^2^: 0%) (Supplementary Figures 9, 10).

###### Occipital Lobe Volume

**FIGURE 7:**
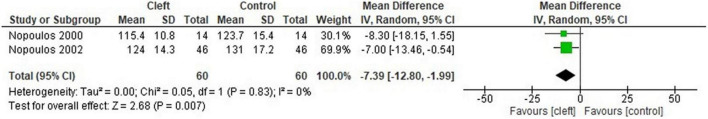
Forest plot for occipital lobe volume (cm^3^).

The cleft group had significantly smaller occipital lobes compared to controls (MD: −7.39 cm^3^; 95% CI: −12.80 to −1.99; *n* = 2; 120 participants; I^2^: 0%) (Figure 7).

Tables 3, 4 summarize studies that were not included in the meta-analyses due to the variability in either methods or outcome.

**TABLE 3:** Regional measurements.

Study	Outcome	Results (mean, SD)
Nopoulos et al. (2000)	Total lobar volumes: frontal, parietal, temporal and occipital	Significantly larger frontal lobes for clefts (440.4, 39.1) than controls (421.4, 46.0; *P* = 0.02). Smaller temporal and occipital lobes for clefts (226.1, 21.7) vs. controls (235.2, 19.9; *P* = 0.02); clefts (115.4, 10.8) vs. control (123.7,15.4; *P* = 0.009), respectfully. No significant differences between parietal lobe volumes.
Nopoulos et al. (2002) and Nopoulos (2002A,B)	Total lobar volumes, gray and white matter volumes provided separately: frontal (and VFC), parietal, temporal (and STP) and occipital	Significantly smaller volumes observed in clefts for all the following: total frontal lobe (463, 55.9) vs. controls (460, 49.7; *P* = 0.029); frontal gray matter (275, 32.3) vs. controls (270, 30.0; *P* = 0.028); parietal lobe (264, 28.0) compared to controls (260, 26.7; *P* = 0.001); parietal gray matter (143, 15.6) vs. controls (139, 15.3; *P* = 0.006); smaller temporal lobe (227, 22.9) vs. controls (238, 20.6; *P* ≤ 0.0001); temporal gray matter (153, 14.4) vs. controls (159, 12.9), *P* = 0.002; temporal white matter (74,0, 10.3) vs. controls (78,9, 10.8; *P* = 0.005); smaller occipital lobe (124, 14.3) vs. controls (131, 17.2; *P* = 0.007); and occipital white matter (Kummer et al., 2007; Mueller et al., 2007; Luo et al., 2018) vs. controls (61.6, 7.39; *P* ≤ 0.0001). The volume of SG (of the VFC) was smaller in clefts (5.876, 1.184) than controls (6.733, 1.533; *P* = 0.02). Total volume of STP greater in clefts (11.96, 1.807) vs. controls (11.61, 1.776; *P* = 0.034), but no significant differences when two sides were compared separately.
Nopoulos et al. (2007c) and Nopoulos (2007A,E)	Lobar gray and white matter volumes separately: frontal (and VFC), parietal, temporal and occipital	*Only means were provided:* Frontal white matter was significantly lower in boys with right clefts (156.0) compared with boys with left clefts (166.3; *P* = 0.01), and healthy boys (164.5; *P* = 0.01). Same was observed occipital white matter in right cleft (35.1), left cleft (39.5) and controls (38.6; *P* = 0.004). The VFC, parietal, temporal lobes, and gray matter of frontal and occipital lobe did not differ between the two groups.
Yang et al. (2012)	STP, thalamus	Total volume of the STP on the left side significantly smaller for cleft subjects (7.42, 2.91) vs. controls (8.77, 3.38; *P* = 0.0006). Thalamus on the left side significantly smaller for cleft (4.98, 0.66) than controls (5.59, 1.06; *P* < 0.001).
Li et al. (2020)	Left postcentral gyrus, right inferior frontal gyrus	*Only narrative data available:* before articulation therapy group had an increased gray matter volume in left postcentral gyrus compared to controls (*P* < 0.001) and after therapy group (*P* < 0.05). Increased gray matter volume in right inferior frontal gyrus in the before therapy group compared to controls (*P* < 0.05).
Weinberg et al. (2013)	Eight corpus callosum landmarks assessed.	Mean corpus callosum shape of cleft subjects was significantly different from controls (Procrustes distance = 0.049; *P* = 0.029). There was a decrease in overall antero-posterior length of the corpus callosum with an increase in convexity of the body in cleft subjects compared to controls.
Nopoulos et al. (2001)	Enlargement of CSP analyzed by a rating scale designed for the study.	One individual out of the 75 controls had an enlarged CSP. Four out of the 49 cleft subjects had enlarged CSP. The incidence of enlarged CSP was significantly different between the two groups (*P* = 0.039).

*VFC, Ventrofrontal cortex; STP, Superior temporal plane; CSP, Cavum septum pellucidum.*

**TABLE 4:** 3D morphometric analysis of brain shape.

Study	Outcome	Results
Nopoulos (2007F)	3D brain shape analyzed with EDMA (interlandmark distances)	*Narrative data:* Major differences in cleft subjects included posterior expansion of the occipital lobe, reorientation of the cerebellum, heightened callosal midbody, and posterior displacement of the caudate nucleus and thalamus. The magnitude of expansion of the occipital lobe was greatest in children with CP.
Weinberg et al. (2009)	3D brain shape analyzed with EDMA (interlandmark distances) and CVA (shape coordinates)	*Narrative data:* Major brain shape changes associated with clefting were observed with CVA and EDMA: this included selective enlargement of the anterior cerebrum coupled with a relative reduction in posterior and/or inferior cerebral portions, changes in the medio-lateral position of the cerebral poles, posterior displacement of the corpus callosum, and reorientation of the cerebellum.

*EDMA, Euclidean distance matrix analysis; CVA, canonical variates analysis; CP, Cleft palate.*

#### Secondary Outcome

##### Studies Investigating Mental and Social Functioning

Heterogeneity of methods and outcomes prevented statistical pooling for meta-analyses for most secondary outcomes, with the exception of IQ scores. These secondary outcomes are illustrated in Table 5.

**TABLE 5:** Psychometric tools used to measure psychosocial functioning.

Study	Outcome	Results	Validated
Nopoulos (2002A)	Social function measured with the Psychiatric Symptoms You Currently have-Baseline tool (PSYCH-base), and the relationship to brain volumes.	Social function was measured only for cleft subjects (recreational interests and activities; relationship with friends and peers; relationship with family members). Twenty-six percent of oral cleft subjects rated relationship with friends as poor. Thirteen percent of oral cleft subjects rated their relationship with family members as poor. Six percent of subjects rated recreational participation as poor. No significant differences of social function between CLP and CP subtypes. Significant correlation was observed between smaller surface of the OF and social dysfunction in cleft subjects (*P* = 0.003).	Yes
Nopoulos (2007B)	Pediatric Behavior Scale derived hyperactivity/impulsivity/inattention (HII) scores and its relationship to the volume of the vmPFC.	The cleft group showed significantly elevated scores in HII compared to controls (*P* = 0.021). Boys of the control group with the lowest right vmPFC volume scored the highest on the HII (*P* = 0.041). In the cleft group, boys with the highest volume of the right vmPFC achieved the highest HII scores (*P* = 0.005).	Yes
Noppulos (2002B)	Boston Naming Test, Rey Auditory-Verbal Learning Test, Rey–Osterreith Complex Figure Test, Stroop Test. Relationship of test performance and brain volumes.	Lower test performance on the Boston Naming Task correlated with greater STP volume for oral cleft subjects, but not significant (*P* = 0.074). No correlations observed in the other tests.	Yes
Bodoni et al. (2021)	RAVEN, Rey Complex Figure, Wisconsin. Relationship between test performance and brain volumes.	Cleft group performed significantly worse on the Raven test compared to controls, and had non-verbal intelligence scores below average (*P* = 0.006). Raven test correlated positively with decreased cortical thickness of right pars orbitalis in oral clefts. Rey Complex Figure Test—Memory scores in oral cleft subjects showed significant positive correlation to decreased cortical thickness in: left supramarginal gyrus, right supramarginal gyrus, left superior parietal lobule, left inferior parietal lobule, right inferior parietal lobule, right middle temporal gyrus, right pars orbitalis, right superior temporal gyrus, and right rostral middle frontal gyrus (*P* ≤ 0.05).	Yes
Nopoulos (2007A)	Self-Description Questionnaire: SDQ-1 and relationship to brain volumes.	Boys with oral clefts had significantly poorer peer relations in the self-reported SDQ-1 score (*P* = 0.002). Significant correlation between small SG measures and self-reported low peer relation scores was observed (*P* ≤ 0.05).	Yes
Nopoulos (2007C)	Speech measured by hypernasality, articulation proficiency, and nasalance. Relationship between performance and brain volumes.	Boys had greater impaired speech than girls in all three domains. These differences reached significance only for the hypernasality rating (*P* = 0.003). Speech and structure correlations for boys with oral clefts were significant for cerebellar volume and articulation (*P* = 0.015), and those with worse articulations had smaller cerebellar volumes.	N/A

*CLP, Cleft lip and palate; CP, Cleft palate; OFC, orbitofrontal cortex; vmPFC, Ventro-medial prefrontal cortex.*

###### Full-Scale IQ

**FIGURE 8:**
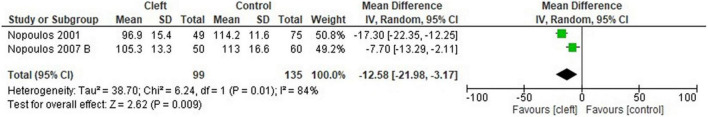
Forest plot for full-scale IQ scores.

Significantly lower FSIQ scores were as observed in individuals with oral clefts compared to controls (MD: −12.58; FSIQ; 95% CI: −21.98 to -3.17; *n* = 2; 234 participants; I^2^ = 84%) (Figure 8). All of the studies used the Wechsler Intelligence Scale of different editions.

### Subgroup Analysis

Four meta-analyses demonstrated moderate to considerable levels of heterogeneity. Subgroup analysis was feasible for only two of the four meta-analyses (Figures 3, 4). Subgroup analyses were performed for age, sex, ethnicity, non-syndromic, and mixed (syndromic and non-syndromic) oral clefts.

#### Total Brain Volume

The non-syndromic subgroup had significantly smaller total brain volume compared to controls. However, this significant difference was not seen in the mixed subgroup (syndromic and non-syndromic cases) (MD: −77.06 cm^3^; 95% CI: −115.47 to −38.64; *n* = 2; 202 participants; I^2^ = 0%; Figure 3). The same phenomenon was observed for age (children vs. adults), sex (male only vs. mixed) and ethnicity (Caucasian vs. mixed) (Supplementary Figures 11A–C). These factors may be possible sources of the heterogeneity seen in the main analysis.

#### Total Cerebral Volume

A decrease in heterogeneity was found in the subgroup analysis of mixed oral clefts (MD: −0.80 cm^3^; 95%CI: −40.88 to 39.29; *n* = 2; 120 participants; I^2^ = 0%; Figure 4). The same phenomenon was observed for age (children vs. adults) and sex (male vs. male and female) (Supplementary Figures 12A,B).

### Reporting Bias

Tests for funnel plot asymmetry could not be used to detect reporting bias due to the few studies included in the meta-analysis (*n* ≤ 10) (Higgins et al., 2019).

## Discussion

The aim of this review was to analyze the empirical evidence of the association between non-syndromic oral clefts and the brain. Overall, oral cleft subjects had smaller cerebral gray matter, cerebellum, temporal lobes, and occipital lobes compared to controls. Individuals with oral clefts had lower FSIQ scores compared to matched controls. Most of the studies controlled for confounders such as age and/or sex to control for brain growth and development; however, only half of the studies for subjects and/or parent’s sociodemographic level (Nopoulos et al., 2000, 2002, 2007c; Li et al., 2020; Bodoni et al., 2021). The risk of bias for the included studies was moderate to high. Most included studies did not analyze cleft subtypes separately which was likely due to the small sample size across subgroups.

Some effects of oral clefts may have remained hidden as a consequence to the small number of studies for most outcomes. A few studies have included syndromic cases of oral cleft, notably Van der Woude syndrome. Van der Woude is a dominantly inherited syndrome caused by the deletion of a gene encoding the interferon regulatory factor-6 (IRF6) on chromosome 1q32 (Johns Hopkins University, 2022). The authors state that the oral cleft occurs in an isolated matter without any other significant developmental issues and allow these individuals to be a part of the non-syndromic group. However, there have been documented cases of cognitive deficits and brain structural abnormalities of Van der Woude syndrome (Nopoulos et al., 2007a; Rincic et al., 2016). Including individuals with Van der Woude syndrome may have an impact on the results of the non-syndromic cleft population.

The total gray matter volume was significantly smaller in the cleft group, an interesting outcome as the total brain and cerebral volume did not significantly differ between the two groups. We hypothesize the following to explain this observation: (1) Shifts in brain tissue distribution in individuals with non-syndromic oral clefts have been shown previously (Nopoulos et al., 2007c). This phenomenon was suggested to occur due to a “compensatory overgrowth” of either brain tissue component unaffecting total brain size (Nopoulos et al., 2002). The cerebellum was significantly smaller in the cleft group; however, the gray or white matter volumes of the cerebellum could not be analyzed separately due to the lack of data in studies. This may indicate the presence of a smaller cerebellar cortex in the oral cleft group (i.e., gray matter), a difference which may not affect the overall tissue size of the “compensated” brain. (2) Subgroup analysis revealed a significantly smaller brain and cerebrum in studies with exclusively non-syndromic oral cleft participants. These differences were not observed in studies with mixed syndromic participants (Figures 3, 4). Total brain gray matter volume was analyzed in studies with non-syndromic individuals exclusively (Figure 2). Non-syndromic oral clefts may have smaller total brain and cerebrum, but the presence of syndromic individuals might have influenced this outcome.

There is supportive evidence regarding a primary unified maldevelopment of the brain during clefting; this might be an underlying etiology for the high risk of neuropsychiatric and neurodevelopmental issues seen in this patient population (Ansen-Wilson et al., 2018). Previous systematic reviews have shown an increased risk of neurodevelopmental and academic difficulties in individuals with non-syndromic oral clefts (Hunt et al., 2005; Al-Namankany and Alhubaishi, 2018; Gallagher and Collett, 2019). These studies, however, highlight the difficulty of summarizing the available evidence due to the lack of uniformity and consistency across studies. It has been proposed that syndromes and additional conditions related to the cleft should be analyzed in a separate group in order to observe if the additional condition is of any way a confounding variable affecting cognitive functioning (Feragen et al., 2014). Future studies should consider the assessment of brain structural data in reference to the subtype of oral clefts, the side affected, additional congenital malformations or comorbidities, anamnestic data on neurodevelopment, age and gender.

Our study has several important limitations. The majority of participants were Caucasian and originated from one register (University of Iowa Cleft Lip and Palate Registry). The clinic-based recruitment and the absence of blinding during the MRI procedures may have introduced bias. Most studies did not report participation rate or investigate the differences between participants and dropouts. We could not analyze structural brain differences across the subtypes of oral cleft and gender due to the small sample sizes. It was not possible to isolate data of the syndromic cases from the overall data of respective studies. Furthermore, the impact of surgical interventions on the developing brain could not be analyzed due to lack of data regarding the timing of the surgery, age of the patient, type of cleft repair surgery and anesthesia exposure. Only one study included the cleft repair status of its participants (Yang et al., 2012). Demographic factors, such as age and/or sex of the participants were provided by most of the included studies; however, there was a lack of detailed information of parental socio-economic factors including education and financial backgrounds. Parental socio-economic factors are known to strongly relate to the child’s neurodevelopment (Noble et al., 2015; Rakesh and Whittle, 2021) and may be a crucial factor in the developing brain of children with oral clefts. It is unclear how brain structural differences affect psychosocial functioning due to the variable assessment tools used in the included studies.

The meta-analyses combined data across studies in order to estimate the effect of oral clefts on brain structure. The main limitations of these meta-analyses are the incomplete reporting of study designs and the variable definition of the patient population across the studies. The interpretation and synthesis of the included studies may have been influenced by these factors. Applicability of our results may be affected due to the limited data for certain subgroups, such as cleft type and gender.

The current review has a number of strengths. To the best of our knowledge, this is the first study to have assessed the overall empirical evidence of brain imaging studies in oral clefts carried out for over two decades. We were able to highlight possible sources of heterogeneity including sex, ethnicity, age and syndromic cases of oral clefts.

There may be structural brain differences between individuals with non-syndromic oral clefts and controls based on the available evidence. Structural brain MRI studies may provide evidence on how the type and degree of clefting plays a role with later cognitive development and functioning. Improvement in study design, size, methodology, and participant selection may allow a more thorough analysis and decrease study heterogeneity. Future studies may greatly benefit the clinical field in establishing timely therapeutic interventions for the necessary cognitive domains as a part of the complex therapy applied to these patients.

## Data Availability Statement

The original contributions presented in the study are included in the article/Supplementary Material, further inquiries can be directed to the corresponding author.

## Author Contributions

KS-B: review design, protocol drafting, search strategy, screening against eligibility criteria, data extraction, data analysis and interpretation, risk of bias assessment, and manuscript drafting. AS: data extraction, data analysis, risk of bias assessment, and manuscript drafting. EV: screening against eligibility criteria, data extraction, and data analysis. GC: search strategy, protocol drafting, data interpretation, and manuscript drafting. GA: review design, protocol drafting, screening against eligibility criteria, data extraction, data analysis, and interpretation. SL: review design, protocol drafting, search strategy, duplicate removals, data analysis and interpretation, risk of bias assessment, and manuscript drafting. All authors contributed to the article and approved the submitted version.

## Conflict of Interest

The authors declare that the research was conducted in the absence of any commercial or financial relationships that could be construed as a potential conflict of interest.

## Publisher’s Note

All claims expressed in this article are solely those of the authors and do not necessarily represent those of their affiliated organizations, or those of the publisher, the editors and the reviewers. Any product that may be evaluated in this article, or claim that may be made by its manufacturer, is not guaranteed or endorsed by the publisher.

## Footnotes

1
https://www.crd.york.ac.uk/prospero/

## References

[B1] Adamson C. L. Anderson V. A. Nopoulos P. Seal M. L. Da Costa A. C. (2014). Regional brain morphometric characteristics of nonsyndromic cleft lip and palate. *Dev. Neurosci.* 36 490–498. 10.1159/000365389 25171633

[B2] Al-Namankany A. Alhubaishi A. (2018). Effects of cleft lip and palate on children’s psychological health: a systematic review. *J. Taibah. Univ. Med. Sci.* 13 311–318. 10.1016/j.jtumed.2018.04.007 31435341 PMC6694901

[B3] Ansen-Wilson L. J. Everson J. L. Fink D. M. Kietzman H. W. Sullivan R. Lipinski R. J. (2018). Common basis for orofacial clefting and cortical interneuronopathy. *Transl. Psychiatry* 8:8. 10.1038/s41398-017-0057-7 29317601 PMC5802454

[B4] Becker D. B. Coalson R. S. Sachanandani N. S. Fair D. Lugar H. M. Kirchner L. E. (2008). Functional neuroanatomy of lexical processing in children with cleft lip and palate. *Plast. Reconstr. Surg.* 122 1371–1382. 10.1097/PRS.0b013e3181881f54 18971720

[B5] Bjørnland T. Nørholt S. E. Rasmusson L. Sándor G. K. (2021). *Nordic Textbook of Oral and Maxillofacial Surgery Munksgaard.* Available online at: https://books.google.hu/books?id=aR1rzgEACAAJ (accessed January 26, 2022).

[B6] Boes A. D. Murko V. Wood J. L. Langbehn D. R. Canady J. Richman L. (2007). Social function in boys with cleft lip and palate: relationship to ventral frontal cortex morphology. *Behav. Brain Res.* 181 224–231. 10.1016/j.bbr.2007.04.009 17537526 PMC1976412

[B7] Bodoni P. S. B. Leoni R. F. do Vale A. B. da Silva P. H. R. Meira Junior S. G. Richieri Costa A. (2021). Neuropsychological functioning and its relationship with brain anatomical measures of children and adolescents with non-syndromic cleft lip and palate. *Child Neuropsychol.* 27 2–16. 10.1080/09297049.2020.1776240 32546116

[B8] Broder H. L. Richman L. C. Matheson P. B. (1998). Learning disability, school achievement, and grade retention among children with cleft: a two-center study. *Cleft Palate Craniofac. J.* 35 127–131. 10.1597/1545-15691998035<0127:LDSAAG<2.3.CO;2 9527309

[B9] Čeponiene R. Hukki J. Cheour M. Haapanen M. L. Ranta R. Näätänen R. (1999). Cortical auditory dysfunction in children with oral clefts: relation with cleft type. *Clin. Neurophysiol.* 110 1921–1926. 10576488 10.1016/s1388-2457(99)00152-2

[B10] Chollet M. B. Nopoulos P. Conrad A. DeLeon V. (2010). Brain morphology of children with cleft lip and/or palate. *FASEB J.* 24 1369–1370.

[B11] Chollet M. B. DeLeon V. B. Conrad A. L. Nopoulos P. (2014). Morphometric analysis of brain shape in children with nonsyndromic cleft lip and/or palate. *J. Child Neurol.* 29 1616–1625. 10.1177/0883073813510603 24381208 PMC4221570

[B12] Cochrane (2020). *Review Manager (RevMan) [Computer program]. Version 5.4.* London: The Cochrane Collaboration.

[B13] Conrad A. L. Canady J. Richman L. Nopoulos P. (2008). Incidence of neurological soft signs in children with isolated cleft of the lip or palate. *Percept. Mot. Skills* 106 197–206. 10.2466/pms.106.1.197-206 18459368 PMC6217843

[B14] Conrad A. L. Dailey S. Richman L. Canady J. Karnell M. P. Axelson E. (2010). Cerebellum structure differences and relationship to speech in boys and girls with nonsyndromic cleft of the lip and/or palate. *Cleft Palate Craniofac. J.* 47 469–475. 10.1597/08-228 20180711 PMC3218570

[B15] Conrad A. L. Wermke K. Eisenmann M. Kuhlmann E. Benavides A. Koscik T. (2021). Preliminary evaluation of pre-speech and neurodevelopmental measures in 7–11-week-old infants with isolated oral clefts. *Pediatr. Res.* 89 85–90. 10.1038/s41390-020-0887-5 32279071 PMC7554202

[B16] DeVolder I. Conrad A. Magnotta V. Nopoulos P. (2015). Difficulties in timing perception related to abnormal brain structure in children and adolescents with nonsyndromic cleft lip and/or cleft palate. *Cleft Palate Craniofac. J.* 52:e110.

[B17] DeVolder I. Conrad A. Richman L. Magnotta V. Nopoulos P. C. (2014). White matter structure in individuals with isolated cleft lip and/or palate: a diffusion tensor imaging study. *Cleft Palate Craniofac. J.* 51.

[B18] DeVolder I. Richman L. Conrad A. L. Magnotta V. Nopoulos P. (2013). Abnormal cerebellar structure is dependent on phenotype of isolated cleft of the lip and/or palate. *Cerebellum* 12 236–244. 10.1007/s12311-012-0418-y 23055082 PMC3566318

[B19] Diaz-Stransky A. Tierney E. (2012). Cognitive and behavioral aspects of Smith-Lemli-Opitz syndrome. *Am. J. Med. Genet. Part C Semin. Med. Genet.* 160C 295–300. 10.1002/ajmg.c.31342 23042585

[B20] Feragen K. B. Stock N. M. Rumsey N. (2014). Toward a reconsideration of inclusion and exclusion criteria in cleft lip and palate: implications for psychological research. *Cleft Palate Craniofac. J.* 51 569–578. 10.1597/12-326 23782417

[B21] Gallagher E. R. Collett B. R. (2019). Neurodevelopmental and academic outcomes in children with orofacial clefts: a systematic review. *Pediatrics* 144:e20184027. 10.1542/peds.2018-4027 31189616

[B22] Hardin-Jones M. Chapman K. (2011). Cognitive and language issues associated with cleft lip and palate. *Semin. Speech Lang.* 32 127–140. 10.1055/s-0031-1277715 21948639

[B23] Higgins J. P. T. Thomas J. Chandler J. Cumpston M. Li T. Page M. J. (2019). “Cochrane handbook for systematic reviews of interventions,” in *Cochrane Handbook for Systematic Reviews of Interventions*, eds Higgins J. P. T. Thomas J. Chandler J. Cumpston M. Li T. Page M. J. (Hoboken, NJ: Wiley).

[B24] Hunt O. Burden D. Hepper P. Johnston C. (2005). The psychosocial effects of cleft lip and palate: a systematic review. *Eur. J. Orthod.* 27 274–285. 10.1093/ejo/cji004 15947228

[B25] Johns Hopkins University (2022). *Online Mendelian Inheritance in Man, OMIM.* Baltimore, MD: Johns Hopkins University.

[B26] Kummer A. W. Lee L. Stutz L. S. Maroney A. Brandt J. W. (2007). The prevalence of apraxia characteristics in patients with velocardiofacial syndrome as compared with other cleft populations. *Cleft Palate Craniofac. J.* 44 175–181. 10.1597/05-170.1 17328642

[B27] Li Z. Zhang W. Li C. Wang M. Wang S. Chen R. (2020). Articulation rehabilitation induces cortical plasticity in adults with non-syndromic cleft lip and palate. *Aging (Albany NY)* 12 13147–13159. 10.18632/aging.103402 32619200 PMC7377881

[B28] Lithovius R. H. Ylikontiola L. P. Harila V. Sándor G. K. (2014). A descriptive epidemiology study of cleft lip and palate in Northern Finland. *Acta Odontol. Scand.* 72 372–375. 10.3109/00016357.2013.840737 24255959

[B29] Luo D. Wan X. Liu J. Tong T. (2018). Optimally estimating the sample mean from the sample size, median, mid-range, and/or mid-quartile range. *Stat. Methods Med. Res.* 27 1785–1805. 10.1177/0962280216669183 27683581

[B30] McDonald-McGinn D. M. Sullivan K. E. Marino B. Philip N. Swillen A. Vorstman J. A. S. (2015). 22q11.2 deletion syndrome. *Nat. Rev. Dis. Prim.* 1:15071. 27189754 10.1038/nrdp.2015.71 PMC4900471

[B31] Mossey P. A. Modell B. (2012). “Epidemiology of oral clefts 2012: an international perspective,” in *Cleft Lip and Palate: Epidemiology, Aetiology and Treatment*, ed. Cobourne M. T. (Basel: S. Karger AG), 1–18. 10.1159/000337464 22759666

[B32] Mueller A. A. Sader R. Honigmann K. Zeilhofer H. F. Schwenzer-Zimmerer K. (2007). Central nervous malformations in presence of clefts reflect developmental interplay. *Int. J. Oral Maxillofac. Surg.* 36 289–295. 10.1016/j.ijom.2006.10.018 17254751

[B33] Muka T. Glisic M. Milic J. Verhoog S. Bohlius J. Bramer W. (2020). A 24-step guide on how to design, conduct, and successfully publish a systematic review and meta-analysis in medical research. *Eur. J. Epidemiol.* 35 49–60. 10.1007/s10654-019-00576-5 31720912

[B34] Noble K. G. Houston S. M. Brito N. H. Bartsch H. Kan E. Kuperman J. M. (2015). Family income, parental education and brain structure in children and adolescents. *Nat. Neurosci.* 18 773–778. 10.1038/nn.3983 25821911 PMC4414816

[B35] Nopoulos P. Berg S. Canady J. Richman L. Van Demark D. Andreasen N. C. (2000). Abnormal brain morphology in patients with isolated cleft lip, cleft palate, or both: a preliminary analysis. *Cleft Palate Craniofac. J.* 37 441–446. 10.1597/1545-15692000037<0441:ABMIPW<2.0.CO;2 11034025

[B36] Nopoulos P. Berg S. Canady J. Richman L. Van Demark D. Andreasen N. C. (2002). Structural brain abnormalities in adult males with clefts of the lip and/or palate. *Genet. Med.* 4 1–9. 10.1097/00125817-200201000-00001 11839951

[B37] Nopoulos P. Boes A. D. Jabines A. Conrad A. L. Canady J. Richman L. (2010). Hyperactivity, impulsivity, and inattention in boys with cleft lip and palate: relationship to ventromedial prefrontal cortex morphology. *J. Neurodev. Disord.* 2 235–242. 10.1007/s11689-010-9060-5 22127933 PMC3164053

[B38] Nopoulos P. Choe I. Berg S. Van Demark D. Canady J. Richman L. (2005). Ventral frontal cortex morphology in adult males with isolated orofacial clefts: relationship to abnormalities in social function. *Cleft Palate Craniofac. J.* 42 138–144. 10.1597/03-112.1 15748104

[B39] Nopoulos P. Richman L. Andreasen N. Murray J. Schutte B. (2007a). Abnormal brain structure in adults with Van der Woude syndrome. *Clin. Genet.* 71 511–517. 10.1111/j.1399-0004.2007.00799.x 17539900

[B40] Nopoulos P. Richman L. Andreasen N. Murray J. C. Schutte B. (2007b). Cognitive dysfunction in adults with Van der Woude syndrome. *Genet. Med.* 9 213–218. 10.1097/GIM.0b013e3180335abd 17438385

[B41] Nopoulos P. Langbehn D. R. Canady J. Magnotta V. Richman L. (2007c). Abnormal brain structure in children with isolated clefts of the lip or palate. *Arch. Pediatr. Adolesc. Med.* 161 753. 10.1001/archpedi.161.8.753 17679656

[B42] Nopoulos P. Berg S. VanDemark D. Richman L. Canady J. Andreasen N. C. (2001). Increased incidence of a midline brain anomaly in patients with nonsyndromic clefts of the lip and/or palate. *J. Neuroimaging* 11 418–424. 10.1111/j.1552-6569.2001.tb00072.x 11677883

[B43] Ornoy A. (2020). Craniofacial malformations and their association with brain development: the importance of a multidisciplinary approach for treatment. *Odontology* 108 1–15. 10.1007/s10266-019-00433-7 31172336

[B44] Page M. J. McKenzie J. E. Bossuyt P. M. Boutron I. Hoffmann T. C. Mulrow C. D. (2021). The PRISMA 2020 statement: an updated guideline for reporting systematic reviews. *BMJ* 372:n71. 10.1136/bmj.n71 33782057 PMC8005924

[B45] Pedersen D. A. Wehby G. L. Murray J. C. Christensen K. (2016). Psychiatric diagnoses in individuals with non-syndromic oral clefts: a danish population-based cohort study Maher B, editor. *PLoS One* 11:e0156261. 10.1371/journal.pone.0156261 27223812 PMC4880322

[B46] Rakesh D. Whittle S. (2021). Socioeconomic status and the developing brain – a systematic review of neuroimaging findings in youth. *Neurosci. Biobehav. Rev.* 130 379–407. 10.1016/j.neubiorev.2021.08.027 34474050

[B47] Richman L. C. Ryan S. M. (2003). Do the reading disabilities of children with cleft fit into current models of developmental dyslexia? *Cleft Palate Craniofac. J.* 40 154–157. 10.1597/1545-1569_2003_040_0154_dtrdoc_2.0.co_2 12605520

[B48] Rincic M. Rados M. Krsnik Z. Gotovac K. Borovecki F. Liehr T. (2016). Complex intrachromosomal rearrangement in 1q leading to 1q32.2 microdeletion: a potential role of SRGAP2 in the gyrification of cerebral cortex. *Mol. Cytogenet.* 9:19. 10.1186/s13039-016-0221-4 26900403 PMC4761178

[B49] Sándor-Bajusz K. Maros T. Olasz L. Sándor G. Hadzsiev K. Vástyán A. (2021). The influence of genetic syndromes on the algorithm of cleft lip and palate repair – a retrospective study. *Ann. Maxillofac. Surg.* 11 270–273. 10.4103/ams.ams_77_21 35265497 PMC8848705

[B50] Scott N. M. Weinberg S. M. Neiswanger K. Brandon C. A. Marazita M. L. (2005). Hair whorls and handedness: informative phenotypic markers in nonsyndromic cleft lip with or without cleft palate (NS CL/P) cases and their unaffected relatives. *Am. J. Med. Genet.* 136 158–161. 10.1002/ajmg.a.30806 15940700

[B51] Shen E. Y. Huang F. Y. (1996). Cleft lip and palate associated with malformation of the central nervous system: a prospective neurosonographic study. *Zhonghua Min. Guo Xiao Er Ke Yi Xue Hui Za Zhi* 37 39–44. 8936009

[B52] Shriver A. S. Canady J. Richman L. Andreasen N. C. Nopoulos P. (2006). Structure and function of the superior temporal plane in adult males with cleft lip and palate: pathologic enlargement with no relationship to childhood hearing deficits. *J. Child Psychol. Psychiatry* 47 994–1002. 10.1111/j.1469-7610.2006.01679.x 17073978

[B53] Tillman K. K. Hakelius M. Höijer J. Ramklint M. Ekselius L. Nowinski D. (2018). Increased risk for neurodevelopmental disorders in children with orofacial clefts. *J. Am. Acad. Child Adolesc. Psychiatry* 57 876–883. 10.1016/j.jaac.2018.06.024 30392629

[B54] Tollefson T. T. Sykes J. M. (2010). Differences in brain structure related to laterality of cleft lip. *Arch. Fac. Plast. Surg.* 12 431–432. 10.1001/archfacial.2010.83 21079123

[B55] van der Plas E. Conrad A. Canady J. Richman L. Nopoulos P. (2010). Effects of unilateral clefts on brain structure. *Arch. Pediatr. Adolesc. Med.* 164 763–768. 10.1001/archpediatrics.2010.123 20679168 PMC3612276

[B56] Veritas Health Innovation (2017). *Covidence Systematic Review Sofware.* Melbourne, VIC: Covidence.

[B57] Wan X. Wang W. Liu J. Tong T. (2014). Estimating the sample mean and standard deviation from the sample size, median, range and/or interquartile range. *BMC Med Res Methodol.* 14:135. 10.1186/1471-2288-14-135 25524443 PMC4383202

[B58] Watkins S. E. Meyer R. E. Aylsworth A. S. Marcus J. R. Allori A. C. Pimenta L. (2018). Academic achievement among children with nonsyndromic orofacial clefts: a population-based study. *Cleft Palate Craniofac. J.* 55 12–20. 10.1177/1055665617718823 34162061

[B59] Wells G. Shea B. O’Connell D. Peterson J. Welch V. Losos M. (2000). *The Newcastle-Ottawa Scale (NOS) for Assessing the Quality of Nonrandomised Studies in Meta-Analyses.* Ottawa, ON: Ottawa Hospital Research Institute.

[B60] Weinberg S. M. Andreasen N. C. Nopoulos P. (2009). Three-dimensional morphometric analysis of brain shape in nonsyndromic orofacial clefting. *J. Anat.* 214 926–936. 10.1111/j.1469-7580.2009.01084.x 19538636 PMC2705301

[B61] Weinberg S. M. Parsons T. E. Fogel M. R. Walter C. P. Conrad A. L. Nopoulos P. (2013). Corpus callosum shape is altered in individuals with nonsyndromic cleft lip and palate. *Am. J. Med. Genet. A* 161A 1002–1007. 10.1002/ajmg.a.35835 23532928

[B62] Yang F. F. McPherson B. Shu H. Xie N. Xiang K. (2012). Structural abnormalities of the central auditory pathway in infants with nonsyndromic cleft lip and/or palate. *Cleft Palate Craniofac. J.* 49 137–145. 10.1597/11-014 21848367

[B63] Zheng W. Li B. Zou Y. Lou F. (2019). The prenatal diagnosis and classification of cleft palate: the role and value of magnetic resonance imaging. *Eur. Radiol.* 29 5600–5606. 10.1007/s00330-019-06089-9 30887208

[B64] Zinkstok J. R. Boot E. Bassett A. S. Hiroi N. Butcher N. J. Vingerhoets C. (2019). Neurobiological perspective of 22q11.2 deletion syndrome. *Lancet Psychiatry* 6 951–960. 10.1016/S2215-0366(19)30076-8 31395526 PMC7008533

