# Supplementary material for: The Brain in Oral Clefting: A Systematic Review With Meta-Analyses
Source: Front Neuroanat. 2022 Jun 10;16:863900. doi: 10.3389/fnana.2022.863900 (PMC9226441; doi:10.3389/fnana.2022.863900)
Supplement: Supplementary file 1 [file Data_Sheet_1.docx]

Supplementary Material

# Supplementary Tables

Supplementary Table 1. Search strategy.

| Database | Date | Search strings |
| --- | --- | --- |
| Scopus | 09.09.2020 | ( ( ( ( ALL ( infant* ) ) OR ( ALL ( adolescent* ) ) OR ( ALL ( children ) ) OR ( ALL ( adult* ) ) OR ( ALL ( boys ) ) OR ( ALL ( girls ) ) OR ( ALL ( males ) ) OR ( ALL ( females ) ) OR ( ALL ( participants ) ) OR ( ALL ( volunteers ) ) OR ( ALL ( subjects ) ) OR ( ALL ( individuals ) ) OR ( ALL ( human* ) ) ) AND ( ALL ( ( ( nonsyndromic OR non-syndromic OR isolated ) AND ( oral OR orofacial ) AND cleft* ) ) ) AND ( ( TITLE-ABS-KEY ( cortical W/3 ( volume OR thickness OR differen* OR development ) ) ) OR ( TITLE-ABS-KEY ( brain W/3 ( structur* OR morphology* OR shape OR dysmorphology* OR morphometr* OR development OR growth ) ) ) OR ( TITLE-ABS-KEY ( ( brain OR cerebrum OR cortex ) W/3 ( abnormal OR differen* ) ) ) ) ) OR ( ALL ( infant* ) ) OR ( ALL ( adolescent* ) ) OR ( ALL ( children ) ) OR ( ALL ( adult* ) ) OR ( ALL ( boys ) ) OR ( ALL ( girls ) ) OR ( ALL ( males ) ) OR ( ALL ( females ) ) OR ( ALL ( participants ) ) OR ( ALL ( volunteers ) ) OR ( ALL ( subjects ) ) OR ( ALL ( individuals ) ) OR ( ALL ( human* ) ) ) AND ( ALL ( ( nonsyndromic OR non-syndromic OR isolated ) AND ( oral OR orofacial ) AND cleft* ) ) AND ( ( TITLE-ABS-KEY ( cortical W/3 ( volume OR thickness OR differen* OR development ) ) ) OR ( TITLE-ABS-KEY ( brain W/3 ( structur* OR morpholog* OR shape OR dysmorpholog* OR morphometr* OR development OR growth ) ) ) OR ( TITLE-ABS-KEY ( ( brain OR cerebrum OR cortex ) W/3 ( abnormal OR differen* ) ) ) ) |
| WOS | 07.09.2020 | ALL= infant* ; ALL= adolescent*; ALL= children; ALL= adult*; ALL= females; ALL= male; ALL= girls; ALL= boys; ALL= individuals; ALL= subjects; ALL= volunteers; ALL= participants; ALL= human*  #1 OR#2 OR #3 OR #4 OR #5 OR #6 OR #7 OR #8 OR #9 OR #10 OR #11 OR #12 OR #13  ALL=((nonsyndromic OR non-syndromic OR isolated) AND (oral OR orofacial) AND cleft*)  AB=(cortical NEAR/3 (volume OR thickness OR differen* OR development) )  AB=(brain NEAR/3 (structur* OR morphology* OR shape OR dysmorphology* OR morphometr* OR development OR growth) )  AB=((brain OR cerebrum OR cortex) NEAR/3 (abnormal OR differen*) )  #18 OR #17 OR #16  #19 AND #15 AND #14 |
| Embase | 07.09.2020 | 1. 'human'/exp 2. 'child'/exp 3. 'infant* 4. 'adolescent* 5. 'children 6. adult* 7. boys 8. girls 9. males 10. females 11. participants 12. volunteers 13. subjects 14. individuals 15. human* 16. #1 OR #2 OR #3 OR #4 OR #5 OR #6 OR #7 OR #8 OR #9 OR #10 OR #11 OR #12 OR #13 OR #14 OR #15 17. 'cleft lip'/exp 18. 'cleft palate'/exp 19. (nonsyndromic OR 'non-syndromic' OR isolated) AND (oral OR orofacial) AND cleft 20. #17 OR #18 OR #19 21. 'brain'/exp 22. (cortical NEAR/3 (volume OR thickness OR differen* OR development)):ti,ab 23. (brain NEAR/3 (structur* OR morphology* OR shape OR dysmorphology* OR morphometr* OR development OR growth)):ab,ti 24. ((brain OR cerebrum OR cortex) NEAR/3 (abnormal OR differen*)):ab,ti 25. #22 OR #23 OR #24 26. #16 AND #20 AND #25 27. #16 AND #20 AND #25 AND [humans]/lim |
| Ovid/Medline | 04.09.2020 | 1. infant*.mp. 2. adolescent*.mp. 3. children.mp. 4. adult*.mp 5. boys.mp. 6. girls.mp. 7. males.mp. 8. females.mp. 9. participants.mp. 10. volunteers.mp. 11. subjects.mp. 12. individuals.mp. 13. human*.mp. 14. Humans/ 15. Adult/ 16. Child/ 17. Adolescent/ 18. Male/ 19. Female/ 20. 1 or 2 or 3 or 4 or 5 or 6 or 7 or 8 or 9 or 10 or 11 or 12 or 13 or 14 or 15 or 16 or 17 or 18 or 19 21. Cleft Lip/ 22. Cleft Palate/ 23. ((nonsyndromic or non-syndromic or isolated) and (oral or orofacial) and cleft*).mp. 24. 21 or 22 or 23 25. (cortical adj3 (volume or thickness or differen* or development)).mp. 26. (brain adj3 (structur* or morpholog* or shape or dysmorpholog* or morphometr* or development or growth)).mp. 27. ((brain or cerebrum or cortex) adj3 (abnormal or differen*)).mp. 28. Cerebrum/ 29. 25 or 26 or 27 or 28 30. 20 and 24 and 29 |
| Cochrane | 07.09.2020 | 1. (infant*) 2. (adolescent*) 3. (children) 4. ((adult*)) 5. ((boys)) 6. ((girls)) 7. ((males)) 8. ((females)) 9. ((volunteers)) 10. ((subjects)) 11. ((participants)) 12. (individuals) 13. (human*) 14. MeSH descriptor: [Male] explode all trees 15. MeSH descriptor: [Female] explode all trees 16. MeSH descriptor: [Age Groups] explode all trees 17. #1 OR #2 OR #3 OR #4 OR #5 OR #6 OR #7 OR #8 #9 OR #10 OR #11 OR #12 OR #13 OR #14 OR #15 OR #16 18. (((nonsyndromic OR non-syndromic OR isolated) AND (oral OR orofacial) AND cleft*)) 19. (brain NEAR/3 (structur* OR morphology* OR shape OR dysmorphology* OR morphometr* OR development OR growth)) 20. ((brain OR cerebrum OR cortex) NEAR/3 (abnormal OR differen*)) 21. (cortical NEAR/3 (volume OR thickness OR differen* OR development)) 22. #19 OR #20 OR #21 23. #17 AND #18 AND #22 |

Supplementary Table 2. Materials used for structural brain MRI analysis. CAT12: Computational Anatomy Toolbox 12; BRAINS: Brain Research: Analysis of Images, Networks, and System.

| Studies | Instrument | Data processing |
| --- | --- | --- |
| Nopoulos et al., 2000 | SIGNA 1.5T MRI | BRAINS |
| Nopoulos et al., 2001 | SIGNA 1.5T MRI | BRAINS |
| Nopoulos et al., 2002 | SIGNA 1.5T MRI | BRAINS |
| Nopoulos et al., 2007 | SIGNA 1.5T MRI | BRAINS |
| Weinberg et al., 2009 | SIGNA 1.5T MRI | BRAINS |
| Yang et al., 2012 | SIGNA 1.5T MRI | FreeSurfer 3.5 |
| Weinberg et al., 2013 | SIGNA 1.5T MRI | BRAINS |
| Adamson et al., 2014 | Siemens TIM Trio 3T MRI | FreeSurfer 5.1.0 |
| Bodoni et al., 2020 | Philips Achieva 1.5T MRI | CAT12 |
| Li et al., 2020 | Siemens TIM Trio 3T MRI | CAT12 |

# Supplementary Figures

Supplementary Figure 1. Forest plot for total brain white matter volume (cm^3^).


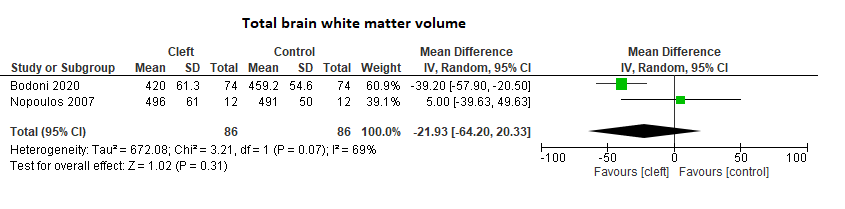


Supplementary Figure 2. Forest plot for cerebral grey matter volume (cm^3^).


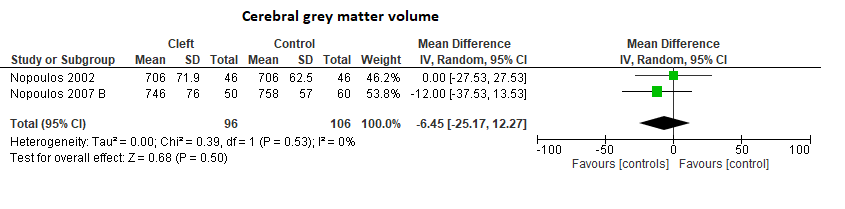


Supplementary Figure 3. Forest plot for cerebral white matter volume (cm^3^).


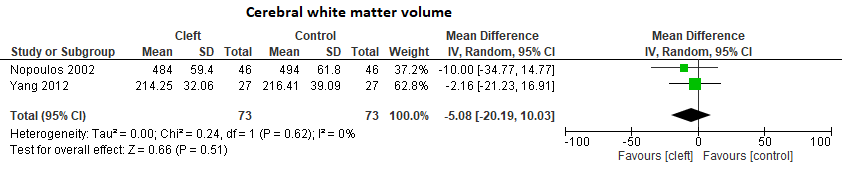


Supplementary Figure 4. Forest plot for frontal lobe volume (cm^3^).


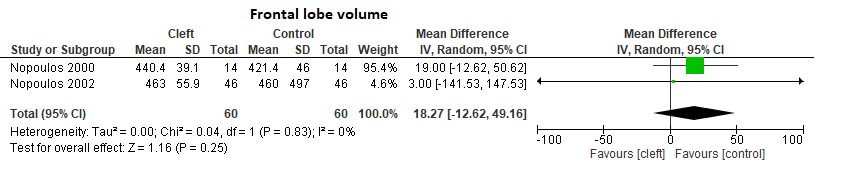


Supplementary Figure 5. Forest plot for frontal lobe grey matter volume (cm^3^).


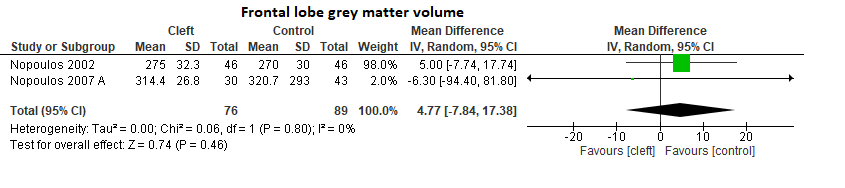


Supplementary Figure 6. Forest plot for straight gyrus (SG) volume (cm^3^).


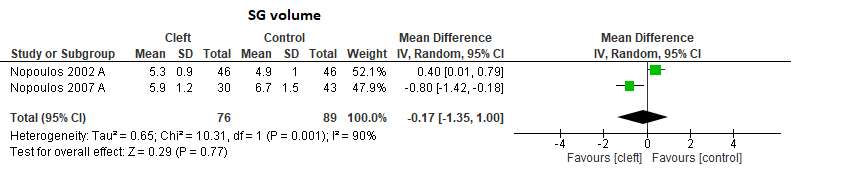


Supplementary Figure 7. Forest plot for orbitofrontal cortex (OFC) volume (cm3).


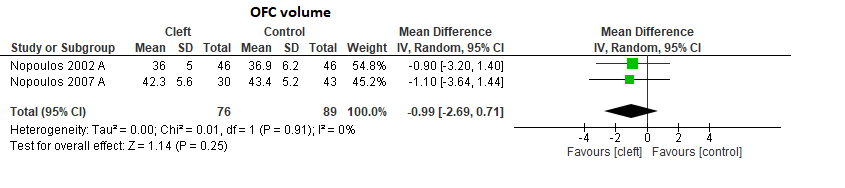


Supplementary Figure 8. Forest plot for parietal lobe volume (cm^3^).


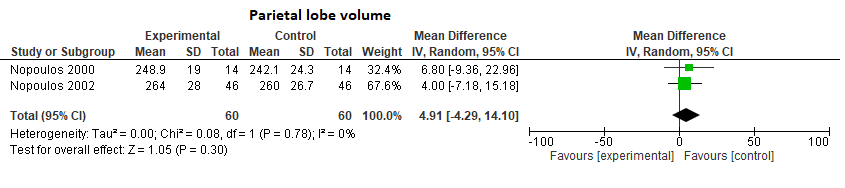


Supplementary Figure 9. Forest plot for superior temporal plane volume, left side (cm^3^).


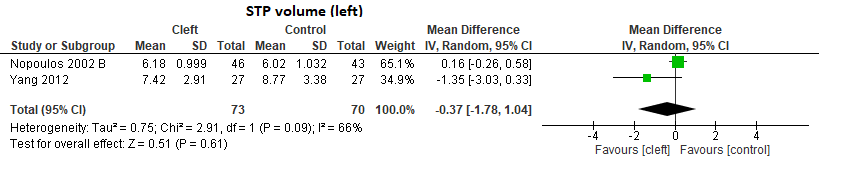


Supplementary Figure 10. Forest plot for superior temporal plane volume, right side (cm^3^).


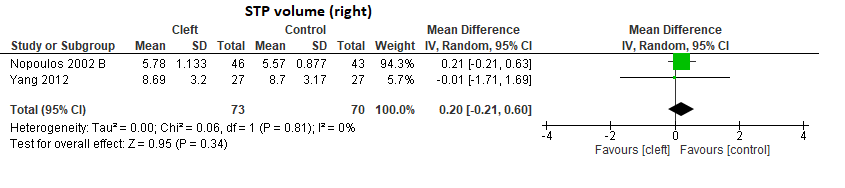


Supplementary Figure 11(A). Forest plot for total brain volume (cm^3^) with subgroup analysis (adults versus children).


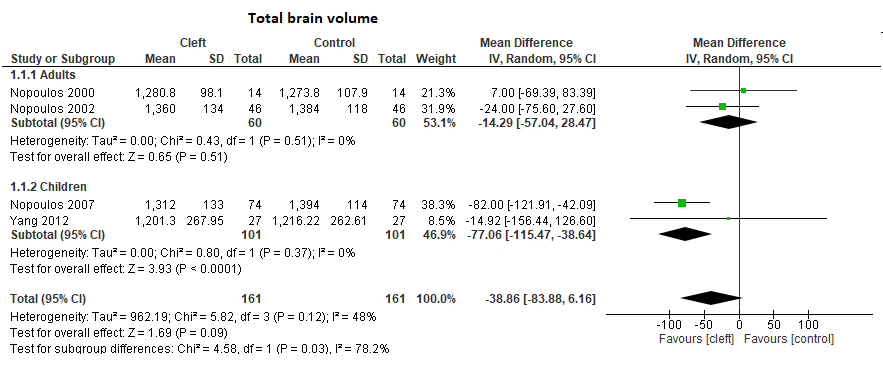


Supplementary Figure 11(B). Forest plot for total brain volume (cm^3^) with subgroup analysis (male versus male and female).


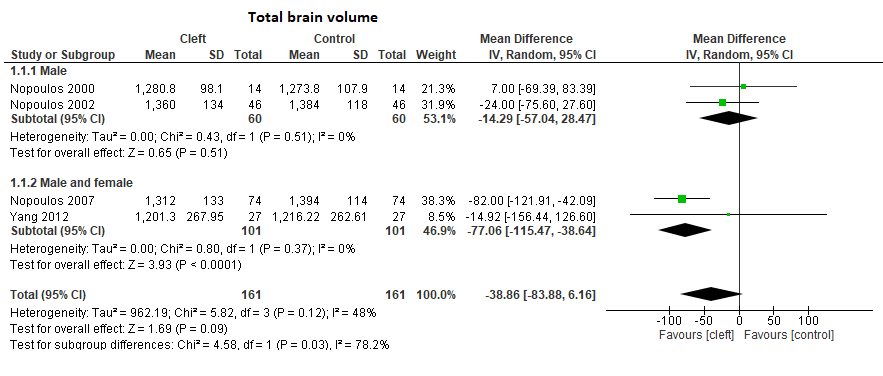


Supplementary Figure 11(C). Forest plot for total brain volume (cm^3^) with subgroup analysis (Caucasian versus mixed).


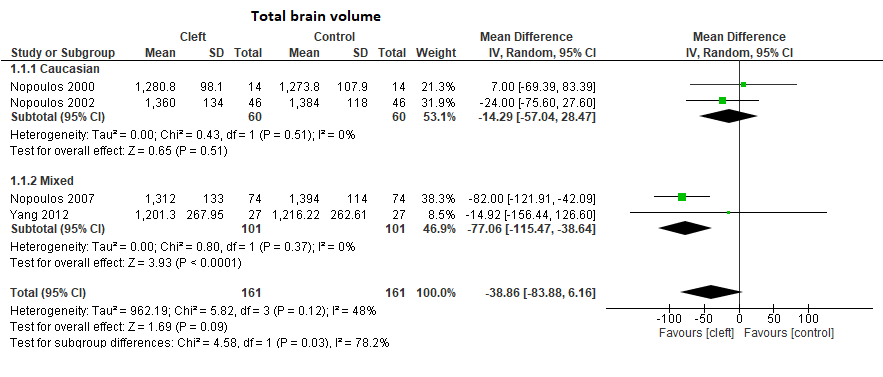


Supplementary Figure 12(A). Forest plot for total cerebral volume (cm^3^) with subgroup analysis (adults versus children).


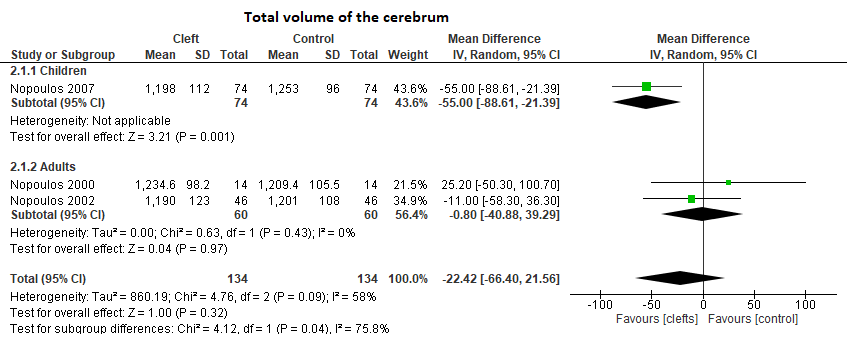


Supplementary Figure 12(B). Forest plot for cerebral volume (cm^3^) with subgroup analysis (male versus male and female).


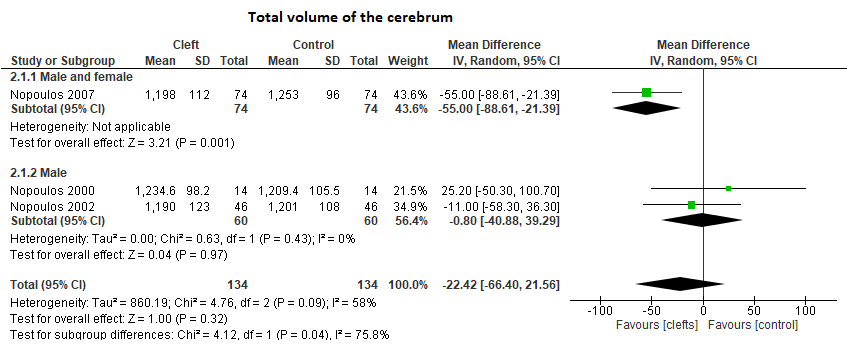


# Dataset repositories: statement

A part of the dataset has been presented on a poster at the 34th ECNP Congress Hybrid, 2-5 October 2021, Lisbon, Portugal. It has been recently published as a conference abstract in the European Neuropsychopharmacology (an Official Journal of the European College of Neuropsychopharmacology).

The citation of the abstract:

P.0201 Brain structure of individuals with oral clefts: first results of a systematic review with meta-analyses. K.A. Sandor-Bajusz, E. Varga, G.N. Antonoglou, S. Lohner. 34th ECNP Congress – Lisbon 2021 Hybrid. DOI:10.1016/j.euroneuro.2021.10.194
